# A multicenter, dose-finding, phase 1b study of imatinib in combination with alpelisib as third-line treatment in patients with advanced gastrointestinal stromal tumor

**DOI:** 10.1186/s12885-022-09610-4

**Published:** 2022-05-06

**Authors:** Maria A. Pantaleo, Michael C. Heinrich, Antoine Italiano, Claudia Valverde, Patrick Schöffski, Giovanni Grignani, Anna K. L. Reyners, Sebastian Bauer, Peter Reichardt, Daniel Stark, Ghimja Berhanu, Ulrike Brandt, Tommaso Stefanelli, Hans Gelderblom

**Affiliations:** 1 Division in Medical Oncology, IRCSS Azienda Ospedaliero-Universitaria di Bologna, Bologna, Italy; 2 Portland VA Health Care System and Oregon Health and Science University, Knight Cancer Institute, Portland, Oregon USA; 3 Institut Bergonie, Bordeaux, France; 4 Hospital Universitario Vall D Hebron, Medical Oncology, Barcelona, Spain; 5 Department of General Medical Oncology, University Hospitals Leuven, Leuven Cancer Institute, and Laboratory of Experimental Oncology, KU Leuven, Leuven, Belgium; 6 Division of Medical Oncology, Candiolo Cancer Institute, FPO – IRCCS, St. Provinciale 142, Km 3.95 - 10060, Candiolo, TO Italy; 7 Department of Medical Oncology, University Medical Center Groningen, University of Groningen, Groningen, The Netherlands; 8 Department of Medical Oncology, Sarcoma Center, West German Cancer Center and German Consortium for Translational Cancer Research (DKTK), University Hospital Essen, Essen, Germany; 9 Department of Oncology and Palliative Care Helios Klinikum Berlin Buch, Berlin, Germany; 10 Leeds Institute for Medical Research, St James’s University Hospital, Leeds, UK; 11 Novartis Pharmaceuticals Corporation, East Hanover, NJ USA; 12 Novartis Pharma AG, Basel, Switzerland; 13 Department of Medical Oncology, Leiden University, Leiden, The Netherlands

**Keywords:** Gastrointestinal stromal tumor, GIST, Alpelisib, Imatinib, Sunitinib, Regorafenib

## Abstract

**Background:**

Acquired resistance to approved tyrosine kinase inhibitors limits their clinical use in patients with gastrointestinal stromal tumor (GIST). This study investigated the safety, tolerability and efficacy of alpelisib, a phosphatidylinositol 3-kinase inhibitor, used in combination with imatinib in patients with advanced GIST who had failed prior therapy with both imatinib and sunitinib.

**Methods:**

This phase 1b, multicenter, open-label study consisted of 2 phases: dose escalation and dose expansion. Dose escalation involved 200 mg once daily (QD) alpelisib, initially, followed by 250 and 350 mg. These were combined with 400 mg QD imatinib until maximum tolerated dose (MTD) and/or a recommended phase 2 dose (RP2D) of alpelisib in combination with imatinib was determined. This MTD/RP2D dose was tested to evaluate the clinical activity of this combination in dose expansion.

**Results:**

Fifty-six patients were enrolled, 21 and 35 in the dose escalation and expansion phases, respectively. The MTD of alpelisib given with imatinib was determined as 350 mg QD. Combination treatment showed partial response in 1 (2.9%) and stable disease in 15 (42.9%) patients. Median progression-free survival was 2 months (95% CI 1.8–4.6). Overall, 92.9% patients had adverse events (AEs) while 46.4% had grade 3/4 AEs, hyperglycemia being the most common (23.2%).

**Conclusions:**

The MTD of alpelisib was estimated as 350 mg QD when used in combination with imatinib 400 mg QD after oral administration in patients with advanced GIST. The safety and tolerability profile of this combination was acceptable; however, the combination did not demonstrate sufficient clinical activity to justify additional clinical testing.

**Trial registration:**

ClinicalTrials.govNCT01735968 (date of initial registration 28/11/2012).

## Background

Gastrointestinal stromal tumors (GISTs) are the most common mesenchymal tumor of the gastrointestinal tract, with a low incidence of 10–15 cases per million people [1, 2]. Activating mutations in either of the 2 receptor tyrosine kinases, KIT (CD117) or the platelet-derived growth factor receptors alpha (PDGFRA), are found in 85% of all GISTs [3, 4]. Gain-of-function *KIT* gene mutations activate downstream signaling pathways (such as phosphatidylinositol 3-kinases [PI3K]), that stimulate cell survival, growth, and proliferation [4], and contribute to tumor development and drug-resistance [5].

Tyrosine kinase inhibitors (TKIs) are the established systemic therapies for KIT-mutated GISTs; imatinib being the first line in patients requiring a systemic therapy [1–4]. Imatinib has demonstrated a median progression-free survival (PFS) of 1.5 years (median follow-up 4.5 years) and median overall survival (OS) of 4.5 years in patients with advanced GIST [6, 7]. Sunitinib, a multi-targeted TKI showing activity against KIT and PDGFRA, is the standard second-line treatment of imatinib-resistant/intolerant GISTs. Similarly, regorafenib is approved in third-line after failure with imatinib and sunitinib [2]. However, both sunitinib [8] and regorafenib [9] achieve a shorter PFS (5–6.5 months) than with first-line imatinib. Recently, ripretinib has been approved (median PFS 6.3 months) for adult patients with advanced GIST who had received prior treatment with 3 or more TKIs (including imatinib, sunitinib, and regorafenib) based on the INVICTUS trial results [10]. Avapritinib is approved for a rare subtype of GIST with a PDGFRA (D842V) mutation [11], but has failed to demonstrate superiority over regorafenib as third-line in unselected GIST patients [12]. In addition, cabozantinib was shown to be active for TKI-resistant GIST in a phase 2 study [13].

Despite the clinical benefits of approved TKIs, acquired resistance to these agents results in fatal disease progression in the majority of patients with advanced GIST [2]. Preclinical studies demonstrated that the PI3K pathway is a crucial survival pathway in imatinib-resistant GIST [14] and some have suggested that the use of TKIs combined with an agent targeting the PI3K pathway may provide long-term benefits in GIST by delaying TKI resistance [1, 15]. The addition of a PI3K inhibitor to imatinib resulted in a prominent decrease of tumor volume and significant antitumor efficacy in a GIST xenograft [16, 17]. We hypothesized that alpelisib, a PI3K inhibitor, combined with imatinib, a KIT inhibitor, might improve outcomes in patients who failed to respond to approved systemic therapies. Of note, no approved treatment for third-line therapy was available when this study (NCT01735968) was planned and initiated.

The current study was conducted to establish the maximum tolerated dose (MTD) and/or a recommended phase 2 dose (RP2D) of alpelisib in combination with imatinib 400 mg once daily (QD) in patients with metastatic and/or unresectable GIST, who had failed prior therapy with both imatinib and sunitinib. The study was then expanded to confirm safety and estimate efficacy.

## Methods

### Study design and treatment

The study consisted of 2 phases: a dose escalation to establish the MTD and/or RP2D, followed by a dose expansion at MTD or RP2D. An adaptive Bayesian Logistic Regression Model with the escalation with overdose control was used in the study. During the dose escalation phase, successive cohorts (3) of newly enrolled patients received increasing doses of alpelisib in combination with imatinib 400 mg QD until MTD and/or RP2D of the combination was determined. The primary objective of the study was to determine the MTD and/or a RP2D of alpelisib when administered orally in combination with imatinib 400 mg QD.

The MTD was defined as the highest drug dosage not expected to cause a dose-limiting toxicity (DLT) in more than 35% of the treated patients in the first cycle of treatment. Defining the MTD required ≥6 patients per dose level. After determination of the MTD and/or RP2D, additional patients were enrolled into the dose expansion phase and treated at the MTD/RP2D to evaluate further the safety and antitumor activity of the combination.

### Patients

Adult patients with a histologically confirmed diagnosis of unresectable or metastatic GIST who had failed prior therapy with imatinib followed by sunitinib, and had World Health Organization performance status of 0–2, were enrolled. Treatment failure was defined as disease progression during therapy (both imatinib and sunitinib) or intolerance to therapy (sunitinib). To enter the dose expansion phase, patients must have had disease progression on both imatinib and sunitinib as documented by ≥1 measurable lesion or confirmation of disease progression by radiological evaluation (computer tomography/magnetic resonance imaging) based on response evaluation criteria in solid tumors (RECIST; 1.1) during prior therapy with imatinib and sunitinib. The patients could have had additional lines of therapy (dose escalation phase) or up to 3 lines of therapy (dose expansion phase).

### Assessments

Adverse events (AEs) were coded using the MedDRA 21.1 while common terminology criteria for AEs (CTCAE; 4.03) was used for grading the severity. Summary of AEs was based on the safety set and focused on the on-treatment period (from day of first dose of study treatment to 30 days after the last dose of study treatment).

The antitumor activity was determined during the dose expansion phase. Tumor response was determined locally by the investigator sites according to Novartis guidelines based on RECIST 1.1. A complete response (CR) or partial response (PR) required confirmation, ≥28 days after first evaluation of response. Efficacy was assessed based on the clinical benefit rate (CBR), overall response rate (ORR), disease control rate (DCR), and PFS. The CBR was defined as proportion of patients with either a best overall response of CR or PR, or a response of stable disease (SD) or better which lasts ≥16 weeks from the start of study treatment (but before progression). The ORR was defined as proportion of patients with the best overall response of CR or PR (confirmed). The DCR was defined as proportion of patients with the best overall response of CR or PR, or a response of SD or better which lasts for ≥6 weeks (but before progression).

### Statistical analysis

The full analysis set (FAS) and safety set consisted of all the patients who received ≥1 dose of alpelisib or imatinib. The dose-determining set (DDS) consisted of patients from the safety set enrolled in the dose escalation phase who either met the minimum exposure criterion and had sufficient safety evaluations (as determined by the investigators and Novartis), or had experienced a DLT during the first cycle (up to day 28 following the start of the combination treatment). The CBR, ORR, and DCR were summarized with the 95% CIs using exact Pearson-Clopper limits. PFS was estimated using the Kaplan-Meier method.

### Ethical Statement

This study protocol was reviewed and approved by the independent ethics committee or institutional review board (Comitato Etico Indipendente del Policlinico Sant’Orsola-Malpighi Padiglione 3, Via Albertoni, 15, Bologna 40138), and the study was conducted according to the ethical principles described in the Declaration of Helsinki. Informed consent was obtained from each participant prior to study entry. The study was registered at clinicaltrials.gov (NCT01735968, date of initial registration 28/11/2012).

## Results

### Patients

Overall, 56 patients with advanced GIST were enrolled in the study (21 in the dose escalation phase and 35 in the dose expansion phase). The baseline characteristics and demographics of patients are described in Table 1. The majority of the enrolled patients had multi-site metastatic disease (Table 1).

**Table 1:** Baseline characteristics and demographics (FAS)

Baseline characteristics	Alpelisib 200 mg + Imatinib (***N*** = 4)	Alpelisib 250 mg + Imatinib (***N*** = 6)	Alpelisib 350 mg + Imatinib (***N*** = 46)	Total (***N*** = 56)
Age, years				
Median (range)	61.5 (47–74)	56.0 (34–67)	59.0 (31–80)	59.0 (31–80)
Age category, n (%)				
< 65 years	2 (50.0)	4 (66.7)	34 (73.9)	40 (71.4)
≥ 65 years	2 (50.0)	2 (33.3)	12 (26.1)	16 (28.6)
Sex, n (%)				
Female	1 (25.0)	1 (16.7)	17 (37.0)	19 (33.9)
Male	3 (75.0)	5 (83.3)	29 (63.3)	37 (66.1)
Race, n (%)				
Caucasian	4 (100)	6 (100)	43 (93.5)	53 (94.6)
Black	0	0	2 (4.3)	2 (3.6)
Pacific Islander	0	0	1 (2.2)	1 (1.8)
Ethnicity, n (%)				
Other	0	3 (50.0)	38 (82.6)	41 (73.2)
Hispanic or Latino	3 (75.0)	0	5 (10.9)	8 (14.3)
Not reported	1 (25.0)	3 (50.0)	3 (6.5)	7 (12.5)
ECOG performance status				
0	2 (50.0)	3 (50.0)	28 (60.9)	33 (58.9)
1	2 (50.0)	3 (50.0)	16 (34.8)	21 (37.5)
2	0	0	1 (2.2)	1 (1.8)
Missing	0	0	1 (2.2)	1 (1.8)
Primary site of cancer				
Small intestine	3 (75.0)	2 (33.3)	21 (45.7)	26 (46.4)
Stomach	0	2 (33.3)	9 (19.6)	11 (19.6)
Peritoneum	1 (25.0)	0	6 (13.0)	7 (12.5)
Abdominal region	0	2 (33.3)	4 (8.7)	6 (10.7)
Omentum	0	0	2 (4.3)	2 (3.6)
Other	0	0	4 (8.7)	4 (7.1)
Site of active disease				
Liver	3 (75.0)	5 (83.3)	31 (67.4)	39 (69.6)
Peritoneum	3 (75.0)	4 (66.7)	28 (60.9)	35 (62.5)
Abdominal region	0	1 (16.7)	23 (50.0)	24 (42.9)
Small intestine	0	2 (33.3)	14 (30.4)	16 (28.6)
Omentum	0	0	11 (23.9)	11 (19.6)
Lung	2 (50.0)	2 (33.3)	7 (15.2)	11 (19.6)
Stomach	0	0	3 (6.5)	3 (5.4)
Large intestine	0	1 (16.7)	2 (4.3)	3 (5.4)
Esophagus	0	0	1 (2.2)	1 (1.8)
Other	3 (75.0)	3 (50.0)	20 (43.5)	26 (46.4)
Prior anti-neoplastic medication – number of regimens				
2	0	2 (33.3)	16 (34.8)	18 (32.1)
3	0	1 (16.7)	20 (43.5)	21 (37.5)
4	1 (25.0)	0	5 (10.9)	6 (10.7)
≥ 5	3 (75.0)	3 (50.0)	5 (10.9)	11 (19.6)
Time since initial diagnosis to first dose of study treatment (months), median (range)	47.38 (18.4–124.2)	69.14 (25.3–151.4)	72.25 (10.4–192.3)	64.66 (10.4–192.3)

*ECOG* Eastern Cooperative Oncology Group, *FAS* full analysis set

All patients (100%) discontinued study treatment, mainly due to progressive disease (64.3%) or AEs (21.4%).

### Maximum tolerated dose

Overall, 19 patients were evaluable for the determination of MTD/RP2D and included in the DDS over 3 cohorts of alpelisib (200 mg, 250 mg, and 350 mg, respectively) in combination with imatinib 400 mg. In cohort 1 (alpelisib 200 mg; *n* = 4), 1 patient experienced a DLT (aspartate aminotransferase increased and blood bilirubin increased), none in cohort 2 (alpelisib 250 mg; *n* = 6), and 2 patients had a DLT (hyperglycemia) in cohort 3 (alpelisib 350 mg; *n* = 9).

### Safety

Adverse events were reported in all patients (100%) at some point during the study. The most common AEs were nausea (66.1%), hyperglycemia (57.1%), diarrhea (53.6%), decreased appetite (39.3%), and vomiting (35.7%). Overall, 75% of patients had grade 3/4 AEs, including hyperglycemia (25%), anemia (12.5%), and abdominal pain (10.7%) as the most common AEs. A total of 92.9% of patients had AEs suspected to be study treatment-related including 46.4% with grade 3/4 AEs. Among these, the most common AE was hyperglycemia (57.1%, all grades; 23.2%, grade 3/4) (Table 2).

**Table 2:** Adverse events suspected to be study treatment-related (overall ≥10%; safety set)

	Alpelisib 200 mg + Imatinib (***N*** = 4)		Alpelisib 250 mg + Imatinib (***N*** = 6)		Alpelisib 350 mg + Imatinib (***N*** = 46)		Total (***N*** = 56)	
	All grades, n (%)	Grade 3/4, n (%)	All grades, n (%)	Grade 3/4, n (%)	All grades, n (%)	Grade 3/4, n (%)	All grades, n (%)	Grade 3/4, n (%)
Total	4 (100)	2 (50.0)	6 (100)	3 (50.0)	42 (91.3)	21 (45.7)	52 (92.9)	26 (46.4)
Hyperglycemia	1 (25.0)	0	1 (16.7)	0	30 (65.2)	13 (28.3)	32 (57.1)	13 (23.2)
Diarrhea	3 (75.0)	1 (25.0)	3 (50.0)	0	21 (45.7)	1 (2.2)	27 (48.2)	2 (3.6)
Nausea	2 (50.0)	1 (25.0)	3 (50.0)	0	21 (45.7)	1 (2.2)	26 (46.4)	2 (3.6)
Decreased appetite	2 (50.0)	1 (25.0)	1 (16.7)	0	12 (26.1)	3 (6.5)	15 (26.8)	4 (7.1)
Vomiting	1 (25.0)	0	1 (16.7)	0	12 (26.1)	0	14 (25.0)	0
Fatigue	0	0	1 (16.7)	1 (16.7)	9 (19.6)	1 (2.2)	10 (17.9)	2 (3.6)
Edema peripheral	0	0	0	0	9 (19.6)	0	9 (16.1)	0
Rash	0	0	0	0	9 (19.6)	1 (2.2)	9 (16.1)	1 (1.8)
Asthenia	2 (50.0)	0	2 (33.3)	0	4 (8.7)	0	8 (14.3)	0
Muscle spasms	0	0	3 (50.0)	0	5 (10.9)	0	8 (14.3)	0
Dysgeusia	0	0	0	0	7 (15.2)	0	7 (12.5)	0
Rash maculo-papular	0	0	0	0	7 (15.2)	2 (4.3)	7 (12.5)	2 (3.6)

Overall, 30 patients (53.6%) had serious adverse events (SAEs) (Table 3). The most common SAEs were anemia, decreased appetite, pyrexia, and renal impairment (5.4%, each). Adverse events led to study treatment discontinuation in 12 patients (21.4%) (Table 3); the most common being decreased appetite, hyperglycemia, and renal impairment (3.6%, each). Out of these 12 patients, AEs were suspected to be study treatment-related in 6 patients. A total of 62.5% of patients had AEs requiring dose interruption and/or change and all the patients required additional supportive therapy for their AEs (Table 3).

**Table 3:** Patients who had grade 3/4 AEs, SAEs, AEs leading to discontinuation, or other significant AEs (safety set)

	Alpelisib 200 mg + Imatinib (***N*** = 4)		Alpelisib 250 mg + Imatinib (***N*** = 6)		Alpelisib 350 mg + Imatinib (***N*** = 46)		Total (***N*** = 56)	
	All grades n (%)	Grade 3/4 n (%)	All grades n (%)	Grade 3/4 n (%)	All grades n (%)	Grade 3/4 n (%)	All grades n (%)	Grade 3/4 n (%)
AEs	4 (100)	4 (100)	6 (100)	5 (83.3)	46 (100)	33 (71.7)	56 (100)	42 (75.0)
Suspected to be study treatment-related	4 (100)	2 (50.0)	6 (100)	3 (50.0)	42 (91.3)	21 (45.7)	52 (92.9)	26 (46.4)
SAEs	1 (25.0)	1 (25.0)	4 (66.7)	3 (50.0)	25 (54.3)	21 (45.7)	30 (53.6)	25 (44.6)
Suspected to be study treatment-related	1 (25.0)	1 (25.0)	0	0	7 (15.2)	6 (13.0)	8 (14.3)	7 (12.5)
AEs leading to discontinuations	1 (25.0)	1 (25.0)	1 (16.7)	1 (16.7)	10 (21.7)	8 (17.4)	12 (21.4)	10 (17.9)
Suspected to be study treatment-related	1 (25.0)	1 (25.0)	0	0	5 (10.9)	3 (6.5)	6 (10.7)	4 (7.1)
AEs requiring dose interruptions and/or change	2 (50.0)	2 (50.0)	3 (50.0)	2 (33.3)	30 (65.2)	22 (47.8)	35 (62.5)	26 (46.4)
Suspected to be study treatment-related	2 (50.0)	2 (50.0)	1 (16.7)	0	22 (47.8)	14 (30.4)	25 (44.6)	16 (28.6)
AEs requiring additional therapy	4 (100)	3 (75.0)	6 (100)	4 (66.7)	46 (100)	29 (63.0)	56 (100)	36 (64.3)
Suspected to be study treatment-related	4 (100)	2 (50.0)	3 (50.0)	0	39 (84.8)	18 (39.1)	46 (82.1)	20 (35.7)

*AE* adverse event, *SAE* serious adverse event

Adverse events of special interest (AESI) were observed in 53 patients (94.6%). For this purpose, similar AEs (and of specific clinical interest) related to alpelisib were grouped under different categories as gastrointestinal toxicity (82.1%), hyperglycemia (57.1%), hypersensitivity and anaphylactic reaction (26.8%), pancreatitis (10.7%), and rash (28.6%).

Overall, 23 deaths (41.1%) were reported during the study, including 12 (21.4%) on-treatment deaths. The majority of the on-treatment deaths were due to underlying malignancy (10 patients, 83.3%); remaining 2 deaths were due to suicide and acute kidney injury, respectively.

### Efficacy

In the dose expansion phase of the study, a PR was observed in 1 patient (2.9%). Fifteen patients (42.9%) had SD as their best overall response. The CBR and DCR were 25.7% (95% CI, 12.5%, 43.3%) and 45.7% (95% CI, 28.8%, 63.4%), respectively (Table 4). For the 5 patients with an unknown response, 3 discontinued due to AEs, 1 due to patient decision and 1 due to progressive disease.

**Table 4:** Best overall response as per investigator review (FAS − expansion phase)

	Alpelisib 350 mg + Imatinib (***N*** = 35)	95% CI
Best overall response, n (%)		
CR	0	
PR	1 (2.9)	
SD	15 (42.9)	
Progressive disease	14 (40.0)	
Unknown^a^	5 (14.3)	
Clinical benefit rate (CBR: CR + PR + SD ≥ 16 weeks)	9 (25.7)	(12.5, 43.3)
Overall response rate (ORR: CR + PR)	1 (2.9)	(0.1, 14.9)
Disease control rate (DCR: CR + PR + SD > 6 weeks)	16 (45.7)	(28.8, 63.4)

^a^Unknown refers to the patients whose post-baseline assessments were not available due to premature treatment discontinuation

*CBR* clinical benefit rate, *CI* confidence interval, *CR* complete response, *DCR* disease control rate, *FAS* full analysis set, *ORR* overall response rate, *PR* partial response, *SD* stable disease

Progression-free survival events were reported in 29 patients (82.9%). Median PFS time was 2 months (95% CI 1.8–4.6) (Table 5).

**Table 5:** Analysis of progression-free survival as per investigator review using Kaplan−Meier method (FAS − expansion phase)

	Alpelisib 350 mg + Imatinib (***N*** = 35)
Number of events, n (%)	29 (82.9)
Progression	24 (68.6)
Death	5 (14.3)
Number of censoring	6 (17.1)
Median PFS time (months), (95% CI)	2 months (1.8, 4.6)

*CI* confidence interval, *FAS* full analysis set, *PFS* progression-free survival

## Discussion

The MTD of alpelisib in combination with imatinib 400 mg QD was determined as 350 mg QD. Median PFS was 2.0 months (95% CI 1.8–4.6). Clinical activity was also observed with one patient (2.9%) achieving PR and 15 patients (43%) having SD as best response. The MTD of alpelisib monotherapy in a first-in-human study was reported as 400 mg QD in patients with solid tumors, which reported a manageable safety profile [18]. In our study, the combination of alpelisib and imatinib (400 mg QD) was used for the first time with a starting dose of alpelisib that was 50% of alpelisib monotherapy MTD (i.e., 200 mg QD).

Imatinib, when used as a monotherapy in patients with GIST who had previous clinical resistance, showed a clinical benefit in only 15% of patients [19–21]. To date, monotherapy with clinically available TKIs is unable to durably overcome resistance caused by secondary KIT/PDGFRA mutations in patients with advanced GIST for longer than 6 months on average, explaining our inability to obtain long-term disease control in the second-line or later lines of therapy. We hypothesized that combination therapy might improve results compared with TKI monotherapy. Alpelisib, a PI3K inhibitor, has shown antitumor activities in combination with other drugs (such as trastuzumab emtansine, fulvestrant, cetuximab, paclitaxel) across patients with a variety of tumors [22–26]. Thus, our study used a combination of imatinib with alpelisib in patients with advanced GIST.

The combination of alpelisib (350 mg QD) and imatinib (400 mg QD) had an acceptable safety profile. Hyperglycemia was the most common AE (57.1%, all grades; 23.2%, grade 3/4). Hyperglycemia is a previously recognized side effect of alpelisib PI3K inhibition [26, 27] and therefore was categorized as an AESI. The incidence of all AESIs was as expected with PI3K inhibitors and alpelisib and was manageable although 4 patients (2 hyperglycemia, 1 rash, and 1 vomiting) discontinued the study treatment due to these AESIs.

Although there are important limitations in comparing across several phase 1 or 2 studies as their results depend upon the details of the cohort and study design selected that may confound the activity results of the agents, our study should be put in context of others, like the INVICTUS trial investigating ripretinib and revealing alopecia as the most common AE (49% patients in ripretinib group; treatment-related) with 9% patients having treatment-related SAEs, while our study showed hyperglycemia as the most common AE (57.1% patients; suspected to be treatment-related) and 12.5% patients with SAEs (suspected to be treatment-related). Also, AEs leading to dose reductions and drug discontinuations were lower in the INVICTUS trial compared to our study (6% vs 10.7%, dose reduction; 5% vs 44.6%, drug discontinuation) [13]. In the GRID trial, regorafenib showed lower incidence of AEs (all grades and grade 3/4) compared to our study (98.5% vs 100%, all grades; 58.3% vs 75%, grade 3/4) [9].

Our results suggest limited clinical activity with only a single PR observed. In the RIGHT trial, a PFS of 1.8 months (95% CI 1.7–3.6) was observed from rechallenge with imatinib after prior imatinib failure in advanced GIST [28] whereas we report alpelisib in combination with imatinib achieved a median PFS of only 2.0 months (95% CI 1.8–4.6). This does not compare favorably with other approved TKI monotherapies such as median PFS of 24.1 weeks (95% CI 11.1–28.3) for second-line sunitinib [8], 4.8 months (95% CI 0.19–0.39) for third-line regorafenib [9], 3.4 months (95% CI 2.4–5.6) for third-line (or further line) pazopanib [29], and 6.3 months (95% CI 4.6–6.9) for fourth-line (or further line) ripretinib [13]. In addition, CABOGIST trial reported a median PFS of 5.5 months (95% CI 3.6–6.9) for cabozantinib in patients with progression after imatinib and sunitinib but no other lines of TKI therapy [13].

A similar study with imatinib (400 mg) in combination with another PI3K inhibitor, buparlisib, in patients with advanced GIST who had failed prior therapy with imatinib and sunitinib showed a median PFS of 3.5 months (95% CI 1.9–5.4) and overall, 98.3% of patients had suspected treatment-related AEs, including 45% with grade 3/4 AEs [30]. However, this combination was not felt to provide additional benefits compared to other existing TKI monotherapy regimens. Additionally, in our study, the combination of alpelisib and imatinib failed to elicit the expected ORR. We hypothesize that these 2 agents may not be acting as a valid biologic combination therapy as all patients had imatinib-resistant GIST. Therefore, our study results may only indicate the single agent activity of alpelisib in this patient population. In a pretreated patient population, PI3K inhibitors may still be relevant, if combined with more potent KIT inhibitors. However, lack of molecular data collection (i.e., KIT and PDGFRA mutation status) is a limitation of this study. Alternatively, there could still be a role for this combination in front-line treatment, but this would require additional clinical investigation.

## Conclusions

The MTD of alpelisib was estimated as 350 mg QD when used in combination with imatinib 400 mg QD after oral administration in patients with advanced GIST. The safety and tolerability profile of this combination was acceptable; however, the combination did not demonstrate sufficient clinical activity to justify additional clinical testing.

## Data Availability

The datasets generated and/or analysed during the current study are not publicly available because Phase 1 studies, by their nature, present a high risk of patient re-identification, but are available from the corresponding author on reasonable request.

## Abbreviations

GIST: Gastrointestinal stromal tumor
PDGFRA: Platelet-derived growth factor receptors alpha
PI3K: Phosphatidylinositol 3-kinase
TKI: Tyrosine kinase inhibitor
PFS: Progression-free survival
OS: Overall survival
MTD: Maximum tolerated dose
RP2D: Recommended phase 2 dose
QD: Once daily
DLT: Dose-limiting toxicity
RECIST: Response evaluation criteria in solid tumors
AEs: Adverse events
CTCAE: Common terminology criteria for AEs
CR: Complete response
PR: Partial response
CBR: Clinical benefit rate
ORR: Overall response rate
DCR: Disease control rate
SD: Stable disease
FAS: Full analysis set
DDS: Dose-determining set
ECOG: Eastern Cooperative Oncology Group
SAEs: Serious adverse events
AESI: Adverse events of special interest
